# To what extent classic socio-economic determinants explain trends of anaemia in tribal and non-tribal women of reproductive age in India? Findings from four National Family Heath Surveys (1998–2021)

**DOI:** 10.1186/s12889-023-15838-x

**Published:** 2023-05-11

**Authors:** Jyoti Ghosal, Madhusmita Bal, Manoranjan Ranjit‬, Arundhuti Das, Manas Ranjan Behera, Sudhir Kumar Satpathy, Ambarish Dutta, Sanghamitra Pati

**Affiliations:** 1 School of Public Health, KIIT Deemed to be University, Bhubaneswar, Odisha India; 2 ICMR-Regional Medical Research Center, Odisha Bhubaneswar, India; 3 Indian Institute of Public Health, Public Health Foundation of India, Odisha Bhubaneswar, India

**Keywords:** Anaemia, Trend, Women of reproductive age group, Tribals, NFHS, India

## Abstract

**Background:**

Despite unprecedented socio-economic growth experienced by Indians in the past few decades, and a long history of anti-anaemia public health measures, prevalence of anaemia in Indian non-pregnant women of reproductive age group (NPWRA) has not declined. This warrants a firm understanding of what explains the anaemia situation over time, preferably by sub-populations. Therefore, we aimed to examine the trends of anaemia in tribal NPWRA (least privileged) and compare with the trends in the NPWRA of general caste (most privileged) between 1998 to 2021. Additionally, the study also explored explanation of any decline and tribal/general narrowing of these trends.

**Methods:**

We studied four rounds of National Family Health Survey (1998–99, 2005–06, 2015–16, 2019–21). We examined the trend of anaemia (haemoglobin < 12 g/dl) and its possible determinants in tribal and general NPWRA and estimated the portion of “decline” and “narrowing” that could be explained by the *underlying* and *intermediate* determinants (wealth, education, residence, parity and food security) using multiple logistic regression.

**Results:**

The distribution of determinants improved over 23 years in both the groups but more in tribals. But anaemia either remained unchanged or increased in both except 7.1 points decline in tribals between 2006–2016, leading also to 7 points narrowing of tribal/general gap. The modest attenuation of beta coefficients representing the change of anaemia prevalence (log of odds) in tribals from -0.314(-0.377, -0.251) to -0.242(-0.308, -0.176) after adjustment with determinants could explain only 23% of the decline. Similarly, only 7% of the narrowing of the tribal/general anaemia gap could be explained.

**Conclusions:**

The structural determinants wealth, education, food security, parity and urban amenities improved immensely in India but anaemia did not decline in this 23-year period. This implies that the “usual suspects” – the structural determinants are not the main drivers of anaemia in the country. The main driver may be absolute and/or functional deficiency status of micronutrients including iron attributable to inadequate uptake and absorption of these elements from Indian diets; and therefore, their effects are noticeable in every socio-economic stratum of India. Future research for aetiologies and new interventions for anaemia alleviation in India may focus on these factors.

## Background

Anaemia is defined as a condition when the number of healthy red blood cells fail to meet the body’s physiological needs for oxygen delivery to vital organs [1–3]. Cognitive and physical capacities of individuals can be seriously compromised by anaemia and can lead to reduced economic productivity and increased morbidity and all-cause mortality [4–8]. Haemoglobin content of the blood is used to measure anaemia. The World Health Organization (WHO) classified adult anaemia as haemoglobin (Hb) levels less than 12.0 g/dL in non-pregnant females and 13.0 g/dL in males respectively [5].

Pathogenesis of anaemia can be explained broadly by three pathways: blood loss, increased destruction of red blood cells and decreased production of red blood cells. Chronic blood loss leading to anaemia are mainly due to helminthic infections, bleeding disorders or abnormal uterine bleeding [9, 10]. Increased destruction of red blood cells occurs either due to structural abnormalities of red blood cells, such as in sickle cell disease or thalassemia, or infections like malaria leading to increased sequestration of red blood cells [11]. Inadequate red blood cell production is either due to increased demand as seen in pregnancy; or chronic infections, malignancies and chronic inflammation [12] which impairs normal bone marrow function; or due to inadequate dietary intake or malabsorption of iron, vitamin A [13], folate, vitamin B12 [14] or protein which are essential ingredients for red blood cell production. More than one of these pathways can co-exist in an individual and all can lead to absolute or functional iron deficiency. Iron deficiency is often portrayed as the most important cause of anaemia, however on some occasions, iron deficiency may be a consequence of any of these three pathways leading to anaemia, rather than being its main driver [5].

Notably, women of reproductive age (WRA) of 15–49 years experience disproportionately higher anaemia compared to men owing to their unique physiology such as menstruation, pregnancy, blood loss during childbirth; and dietary inequity within the household [15]. Anaemia among WRA has far-reaching impact not only on the health of the women but also on the progeny, one of the leading pathways to inter-generational ill health [16] and poverty in anaemia-ravaged populations [6, 17].

Anaemia substantially contributes to the global burden of ill-health [18, 19]. It affected 1.8 billion individuals worldwide [20] accounting for 50.3 million years lived with disability in 2019; [21] globally 30.1% of WRA were anaemic in 2019, [22] with wide geographical variations. Because of the scale of the anaemia problem [23, 24], in May 2012, the 65^th^ World Health Assembly (WHA) endorsed a Global Nutrition Target (GNT) to reduce anaemia in WRA by 50% by 2025 [25]; which has since been extended to 2030 as proposed by WHO and UNICEF to be aligned with UN Sustainable Development Goal (SDG) [26]. In October 2019, the prevalence of anaemia in WRA was incorporated as an indicator 2.2.3 under the target 2.2 of the 2nd goal of SDGs [26, 27].

South Asia is the largest contributor to the global anaemia burden [21], 49% of WRA of this region were found to be anaemic in 2019 [28]. India, the largest South Asian nation, had been experiencing high prevalence of anaemia since time immemorial. Even currently, anaemia continues to plague the nation despite its tremendous economic development over the past few decades and all the anti-anaemia public health programmes it has mounted at the problem [29].

This recalcitrance of anaemia in India in the face of its high economic growth is very intriguing. In this backdrop, studying anaemia in vulnerable, underprivileged sub-populations can provide us with the necessary insight into the problem, because the underprivileged are most likely to experience higher levels of anaemia, but at the same time their anaemia burden may show signs of improvement in contrast to the stagnant national trends, because of the so-called “base effect”. The tribal (constituting 8.6% of India’s population) non-pregnant WRA (NPWRA) of India, who are amongst the most underprivileged in the nation and predisposed to anaemia, represents such a vulnerable study population [30].

Therefore, we aimed to examine the trends of anaemia in the tribal NPWRA and compare with the trends in the NPWRA of the general caste – the most privileged sub-population of India – at four time points spanning over 23 years (1998 to 2021). Additionally, the study also explored explanation of any decline and tribal/general narrowing of these anaemia trends.

## Methods

### Data

The study employed a repeated cross-sectional design analysing data from four rounds of National Family Health Surveys—2 (NFHS-2, 1998–99), 3 (NFHS-3, 2005–06), 4 (NFHS-4, 2015–16) and 5 (NFHS-5, 2019–2021). NFHS is the Indian version of periodic Demographic Health Survey (DHS) which are conducted in almost every country worldwide on nationally representative sample of WRA, using harmonized formats with the purpose of cross-nation comparisons [31].

NFHS-2 covered 91,196 households and interviewed 90,303 WRA from all the then 26 Indian states (did not cover any union territories) in 1998–1999. A multi-stage clustered sampling design was used to select the household and respondents. Household and eligible women response rate in NFHS-2 was 97.5% and 95.5%. Seven years later, 3^rd^ round of survey took place where 109, 041 households were covered and 124,385 WRA from all 29 states were interviewed with 97.7% and 94.5% household and eligible women response rates. Subsequently, a decade later, using a similar sampling design NFHS-4 covered 601,509 households and interviewed 699,686 WRA from 29 states and 7 union territories having household and eligible women response rates as 97.6% and 96.7% respectively. In 2019–2021 NFHS-5 covered 636,699 households and interviewed 724,115 WRA from all states and Union territories. Household and eligible women response rates in NFHS-5 was 98% and 97% [31]. Data in all the rounds of NFHS were collected from the women respondents as well as the head of households.

**Fig. 1:**
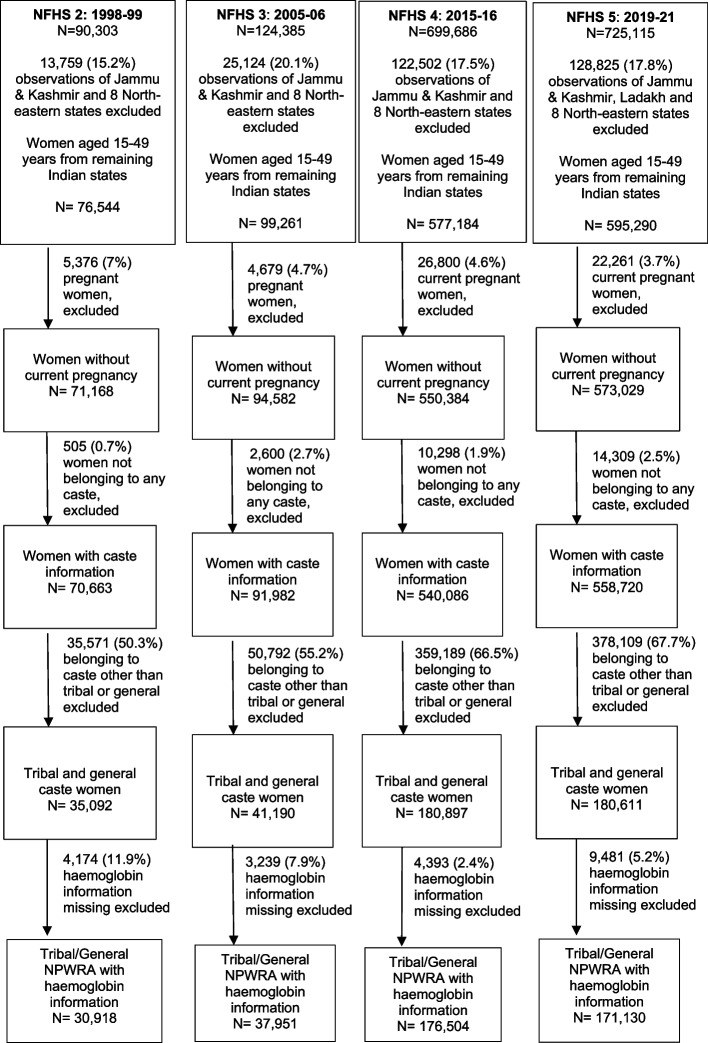
A schematic flow chart of the study sample across the four rounds (2, 3, 4, 5) of National Family Health Survey

Only non-pregnant WRA (NPWRA) belonging to ST and general caste were included in our analyses. As these two sub-populations represent the two extremes of the social privilege spectrum in India—the ST being the most marginalized and the general caste being the least—it was assumed that the inclusion of these extreme groups would help to compare and contrast the trend of anaemia of STs more comprehensively. Jammu & Kashmir, Ladakh (in NFHS-5) and 8 north-eastern states—Arunachal Pradesh, Assam, Manipur, Meghalaya, Mizoram, Nagaland, Sikkim, and Tripura were excluded from our analyses because the tribes residing in these states/UTs are privilege-wise much closer to the mainstream as opposed to tribal peoples from other parts of India [31]. The study samples are schematically summarized in Fig. 1.

### Variables

Anaemia status was the outcome in this study. Haemoglobin was measured from the capillary blood collected from the women and tested using HemoCue Photometer system [31]. Haemoglobin levels less than 12 gm/dl was termed as anaemia (yes/no) as per WHO definition of anaemia for non-pregnant women [5]. The tribal or caste identity of the women was considered based on self-reported membership by the head of the household at the time of interview. A composite wealth index, later quintiled, was constructed from multiple household assets (assets varied between rounds of NFHS), access to water and sanitation facilities, household fuel use and landholding patterns using principal component analysis. These asset variables were also reported by the head of the household. Body Mass Index (BMI) was calculated from objectively-measured weight and height (kg/m^2^) and was categorised into binary variable: underweight (< 18.5 kg/m^2^); underweight (‘undernutrition’ was used interchangeably with ‘underweight’ in the article) was a proxy indicator of household food security. Education was measured as completed years of formal education. Place of residence was a binary variable (rural/urban) and was a proxy indicator of absence or presence of urban amenities. Here, rural denoted any place which had a population less than 5,000, density of population less than 400 per square km and more than 25% of the male working population is engaged in agricultural pursuits. Age in completed years was recorded and then dichotomized: 15–30 and 31–49 years.

Age, wealth quintile, education, place of residence, number of children born and body mass index of NPWRA were used as categorical variables for descriptive statistics and were used as continuous variable in subsequent regression models.

### Co-variate selection

One of our main research aims was to examine what explains the decline of anaemia among tribal and narrowing of the tribal/general anaemia gap. Therefore, we reviewed several existing conceptual models of the anaemia causation cascade [6, 32–34] to identify the determinants of anaemia systematically. The determinants were broadly classified into three levels: *underlying*, *intermediate*, and *immediate*. We explored the roles of *underlying* (wealth, education, place of residence, and parity); and *intermediate* determinants (food security) of anaemia, because in every discourse, as evident from the existing conceptual frameworks, they garner all the attention as the “usual suspects” [6, 35]. Therefore, we planned for a closer empiric scrutiny of the roles of these structural determinants.

### Statistical analysis

Prevalence of anaemia was estimated for the tribal and general NPWRA for each round of NFHS and plotted. Between rounds 3 (2006) and 4 (2016) we could observe a substantial decline only in tribal anaemia, bringing their estimates closer to the general group, however, there was no decline and therefore, tribal/general narrowing in anaemia during other periods.

First, we examined the differences in distribution of the *underlying* and *intermediate* determinants across the two sub-populations using cross-tabulation and chi square test – separately for each of the four rounds. Then, we studied association between the identified determinants and outcome in a dataset pooled for all the four rounds, using multi-variable logistic regression and the identifier for round introduced as a dummy variable to adjust for *between-round* heterogeneity– Odds Ratios (OR) with *p*-value and 95% Confidence Interval used as an estimate of independent significant association. We found age, wealth quintile, education, place of residence, number of children born and body mass index of NPWRA to be independently associated with their anaemia status (p < 0.05). We also included the quadratic terms for age (age^2^) and BMI (BMI^2^) to explore their non-linear relationship, if any, with anaemia. However, the quadratic terms hardly improved the model fit, the metric of goodness-of-fit pseudo R squared, only changing very marginally after inclusion of BMI^2^ and age^2^. Therefore, we decided not to include the quadratic terms in our explanation models which are described below.

**Fig. 2:**
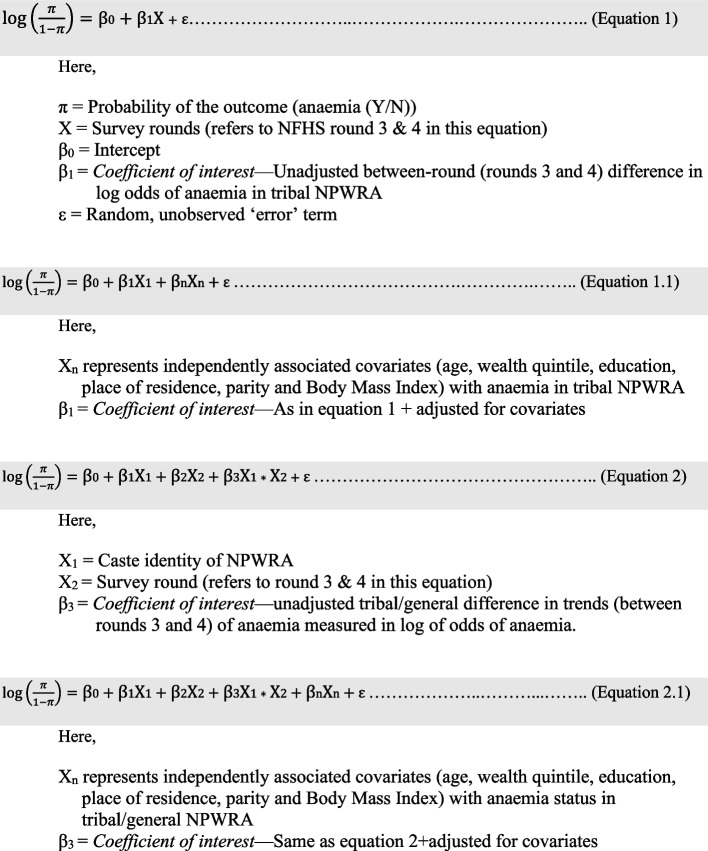
Mathematical equations of the explanatory models

We set out to estimate the portion of decline in tribal anaemia (between 2006 and 2016) that could be explained by the *between-round* changes in these independently associated determinants in the tribals during this period. For that we first used data pooled for rounds 3 and 4, but only for tribals. Using logistic regression model the unadjusted decline in anaemia between these two rounds in the tribal group was estimated (Fig. 2: Eq. 1)—beta coefficient of the “round” variable representing the unadjusted difference of average log of odds of tribal anaemia.

Then the model was adjusted for the independently associated variables (Fig. 2: Eq. 1.1) and the percentage change in beta coefficients represented the portion of anaemia decline explained by changes in these determinants among tribals.

Decline in anaemia only in tribal and not in general meant narrowing of the tribal/general anaemia gap between these two rounds. This afforded us an opportunity to study the same phenomenon of decline of anaemia only among tribals from a different angle. We now set out to measure the portion of this narrowing that could be explained by the changes in *between-group* and *between-round* distribution of these determinants. Again, using logistic regression model we estimated the unadjusted narrowing of the anaemia gap in a dataset that included both tribal and general NPWRA for rounds 3 and 4 pooled together, through the beta coefficient of the interaction term: caste*round (Fig. 2: Eq. 2).

The model was further adjusted for the independently associated determinants of anaemia (Fig. 2: Eq. 2.1). The change in the beta coefficient of the interaction terms represented the portion of narrowed anaemia gap explained by the changes in *between-group* and *between-round* distribution of these determinants.

STATA version 15.1 [36] and R version 4.1.2 [37] were used to conduct analyses. Cluster sampling design and survey sampling weights were used for all estimation processes.

## Results

The study included 30,918 (NFHS-2), 37,951 (NFHS 3), 176,504 (NFHS 4) and 171,130 (NFHS 5) non-pregnant women of reproductive age (NPWRA) (Fig. 1). The educational attainment improved, urban residence increased, parity decreased, and undernutrition improved in both the groups, tribal and general NPWRA, over all the four rounds. But we could not comment on temporal changes of absolute amount of wealth as wealth was measured using different scales in different rounds.

### Temporal changes in distribution of determinants

However, in every round the tribal women were more rural, with less education, more likely to be undernourished and bearing greater number of children than their general caste counterparts (Table 1). The tribal NPWRA consistently belonged to poorer quintiles of wealth in greater proportions than general.

**Table 1:** Trends of identified determinants of anaemia in Indian tribal and general caste non-pregnant women of reproductive age using National Family Health Surveys (NFHS) from 1998–2021

**Determinants of anaemia**	**NFHS-2 (1998–1999)**		***p*-Value**	**NFHS-3 (2005–2006)**		***p*-Value**	**NFHS-4 (2015–2016)**		***p*-Value**	**NFHS-5 (2019–2021)**		***p*-Value**
	**General (%)**	**Tribals (%)**		**General (%)**	**Tribals (%)**		**General (%)**	**Tribals (%)**		**General (%)**	**Tribals (%)**	
**Age**	***N* = 25,412**	***N* = 5,505**		***N* = 30,423**	***N* = 7,528**		***N* = 127,497**	***N* = 49,007**		***N* = 119,081**	***N* = 52,049**	
15–30 years	46.2	53.7	< 0.001	55.1	58.4	< 0.001	51.3	56.1	< 0.001	49.3	54.2	< 0.001
31–49 years	53.8	46.3		44.9	41.6		48.7	44		50.7	45.8	
**Place of residence**	***N* = 25,412**	***N* = 5,505**		***N* = 30,423**	***N* = 7,528**		***N* = 127,497**	***N* = 49,007**		***N* = 119,081**	***N* = 52,049**	
Urban	35.0	10.8	< 0.001	43.2	10.1	< 0.001	46.6	14.9	< 0.001	43.5	13.9	< 0.001
Rural	65.0	89.2		56.8	89.9		53.4	85.1		56.5	86.1	
**Education (years)**	***N* = 25,398**	***N* = 5,505**		***N* = 30,418**	***N* = 7,527**		***N* = 127,497**	***N* = 49,007**		***N* = 119,081**	***N* = 52,049**	
Illiterate	37.8	79.3	< 0.001	24.7	65.8	< 0.001	15.7	45.3	< 0.001	13.1	37.4	< 0.001
1–8	34.4	15.8		31.1	21.8		26.9	27.4		25.1	28.4	
9–10	14.3	3.2		20.8	8.1		21.5	14.9		22.2	17.2	
11–12	6.1	1.0		10.5	2.8		15.4	7.5		15.9	9.8	
Above 12	7.4	0.7		12.9	1.5		20.5	4.9		23.7	7.2	
**Wealth quintile**	***N* = 25,412**	***N* = 5,505**		***N* = 30,423**	***N* = 7,528**		***N* = 127,497**	***N* = 49,007**		***N* = 119,081**	***N* = 52,049**	
5^th^ (Richest)	23.8	2.5	< 0.001	24.4	2.4	< 0.001	26.5	3.1	< 0.001	27.2	3.5	< 0.001
4^th^	23.1	5.8		23.8	4.5		24.8	8		25.4	7.7	
3^rd^	21.5	12.9		22.7	8.9		22.4	13.9		22.2	15.0	
2^nd^	18.2	28.3		18.6	25.8		17.4	26.7		16.7	27.5	
1^st^ (Poorest)	13.4	50.5		10.5	58.4		8.9	48.7		8.5	46.3	
**Number of children born**	***N* = 25,412**	***N* = 5,505**		***N* = 30,423**	***N* = 7,528**		***N* = 127,497**	***N* = 49,007**		***N* = 119,081**	***N* = 52,049**	
0	8.3	10.9	< 0.001	29.3	26.2	< 0.001	30.0	28.9	< 0.001	29.6	30.4	< 0.001
1	14.3	12.3		12.4	9.7		15.4	12.1		16.1	12.0	
2	25.4	17.2		23.8	14.9		28.9	22.1		31.2	23.9	
3	20.9	19.0		15.8	16.4		14.4	17.2		13.6	17.4	
> 3	31.1	40.5		18.7	32.8		11.3	19.7		9.5	16.3	
**Undernutrition**	***N* = 25,292**	***N* = 5,477**		***N* = 30,380**	***N* = 7,515**		***N* = 127,229**	***N* = 48,890**		***N* = 118,791**	***N* = 51,935**	
Yes	30.8	50.2	< 0.001	29.1	50.5	< 0.001	17.5	34.1	< 0.001	14.8	27.4	< 0.001

Between rounds 3 and 4, poverty gap improved (measured by proportion in poorest quintile 47.9 points to 39.8 points), illiteracy gap improved (41.1 points to 29.6 points), parity gap improved (measured by proportion having > 3 children from 14.1 points to 8.4 points) and undernutrition gap improved (from 21.4 points to 16.6 points), because these indicators among the tribals improved at a greater rate than the improvement in general caste. Such considerable improvements in gaps were not observed between rounds 2 and 3 as well as between 4 and 5.

### Association between identified determinants and anaemia

Additionally, the multi-variable logistic regression further confirmed age, wealth, education, place of residence, number of children born and body mass index of NPWRA to be independently associated with their anaemia status. (Table 2).

**Table 2:** Associations of framework guided determinants of anaemia (exposure) with anaemia (outcome) among non-pregnant women of reproductive age group in India using pooled data from 1998 and 2021 of National Family Health Surveys (NFHS)

Determinants of anaemia (*N* = 415,965)	Odds Ratio	*p*-Value	95% Confidence Interval (CI)	
			**Lower CI**	**Upper CI**
Age (years)	0.9975	< 0.001	0.9962	0.9989
Caste identity (Reference: General)	1.3113	< 0.001	1.2814	1.3420
Place of residence (Reference: Urban)	0.9717	0.02	0.9483	0.9955
Education (years)	0.9903	< 0.001	0.9880	0.9926
Wealth Index (quintile) (Reference: 1^st^ quintile is the poorest)	0.9080	< 0.001	0.8997	0.9163
Number of children born	1.0141	< 0.001	1.0071	1.0212
Body Mass Index (BMI)	0.9998	< 0.001	0.9998	0.9998

### Trend of anaemia

**Fig. 3:**
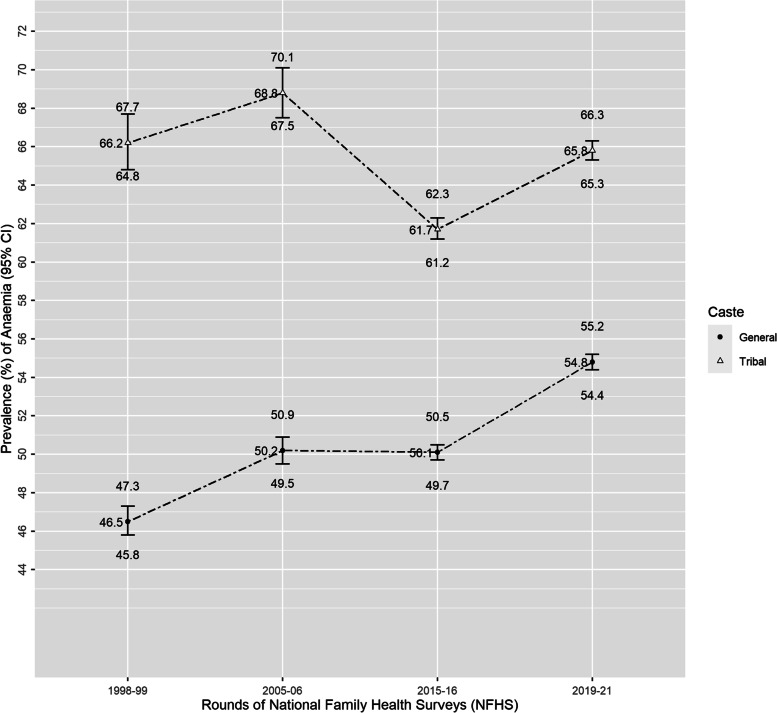
Trend of anaemia among Indian tribal and general caste non-pregnant women of reproductive age using National Family Health Surveys (NFHS) from 1998–2021

Anaemia was highly endemic in both the groups during the four rounds of survey. The prevalence of anaemia among NPWRA of general caste had modestly increased from 46.5% in NFHS-2 to 54.8% in NFHS-5, remaining constant around 50% in rounds 3 and 4. Tribal anaemia tracked almost parallelly with anaemia in general NPWRA between rounds 2 and 3 (tribal/general gap 19.7 in NFHS-2 and 18.6 in NFHS-3) and between 4 and 5 (11.6 points in NFHS-4 and 11 points in NFHS-5) (Fig. 3). The substantial decline of 7.1 points in tribal anaemia between rounds 3 and 4 narrowed the tribal/general gap also (18.6 points in NFHS3 and 11.6 points in NFHS4).

### Explained portion of decline of tribal anaemia between rounds 3 and 4

The unadjusted beta coefficient of “rounds” (representing the decline of anaemia measured in log of odds of anaemia) attenuated from -0.314 (-0.377, -0.251) to -0.242 (-0.308, -0.176) after adjusting for the selected independently associated determinants. Therefore, only 23% of the tribal anaemia decline could be explained by the between-round changes in the distribution of those independently associated determinants (Table 3).

**Table 3:** Portion of Indian tribal anaemia decline explained along with narrowing of tribal/general anaemia gap between 2005–2016 of National Family Health Surveys (NFHS)

**Anaemia**	**Decline in log of odds of anaemia between NFHS rounds 3 & 4 in tribal NPWRA** **(95% CI)**	**Portion of decline explained (%)**
Unadjusted (*N* = 70,390)	-0.314 (-0.377, -0.251)	23%
Adjusted^a^ (*N* = 70,335)	-0.242 (-0.308, -0.176)	
**Anaemia**	**Narrowing of tribal/general anaemia gap measured in log of odds of anaemia between NFHS rounds 3 & 4** **(95% CI)**	**Portion of narrowing explained (%)**
Unadjusted (*N* = 214,455)	-0.311 (-0.382, -0.241)	7%
Adjusted^a^ (*N* = 214,263)	-0.288 (-0.358, -0.217)	

^a^Model was adjusted with age, place of residence, education, wealth, number of children born, body mass index

### Explained portion of narrowing of tribal/general anaemia gap between rounds 3 and 4

During the same period, beta coefficient of the “caste*round” interaction term (representing the narrowing of tribal/general anaemia gap measured in log of odds of anaemia) attenuated from -0.311 (-0.382, -0.241) to -0.288 (-0.358, -0.217) after adjusting for the independently associated variables. Therefore, only 7% of the narrowing of the tribal/general anaemia gap could only be explained by the changes in *between-group* and *between-round* distribution of these determinants (Table 3).

## Discussion

Between 1998 and 2021, India experienced hardly any reduction of anaemia prevalence among non-pregnant reproductive-age group women (NPWRA). Not only the overall prevalence of anaemia remained persistently high during these 23 years, but it also increased modestly during this period, 51.8% in 1998 to 57.2% in 2021 [31]. This was when India experienced remarkable economic development, moving from the command economy to free market economy in the 1990s [38]. Per capita Gross National Income (constant 2015 international) of India increased from 690.5 USD in 1998 to 1,930.7 USD in 2021 [39]. This economic development positively impacted many important social indicators, which are also determinants of anaemia. For instance, in our study, we observed notable improvement in food security (measured by proportion of underweight adults), increasing educational attainment, rising urbanisation, and declining parity over these 23 years. Despite the talk of inequitable development in India [40], our data also showed that even one of the most disadvantaged groups such as the tribals also benefitted immensely from this economic growth, as all their indicators improved substantially. But all this development could not bring down anaemia considerably in almost any sub-population of Indian NPWRA.

The popular conceptual frameworks [6, 32–34] posit wealth as a principal underlying determinant of anaemia because anaemia is economically patterned. So, alleviation of poverty should have led to anaemia reduction in Indian NPWRA. But in reality, despite tremendous poverty alleviation in India in the first two decades of the twenty-first century [38, 40], there was no noticeable decline in the prevalence of anaemia in Indian NPWRA. Moreover, anaemia continued at high rates in every section of the country, rich or poor. This indicates that wealth as well as economic inequality of anaemia are not its main drivers in the Indian context, albeit anaemia remined socio-economically patterned in the Indian society throughout the period [41]. In the above example, instead of wealth if one considers the role of education, one will see that improvements in educational levels also have hardly impacted anaemia in India. Therefore, only focusing on reducing structural determinants such as poverty and illiteracy— the ‘usual suspects’ [42], also referred to as the “causes of causes”—will not solve the anaemia problem of India. Therefore, the main driver of anaemia in India is likely to be absolute and/or functional deficiency of micronutrients including iron– the *immediate* determinant of anaemia. But in India, these insufficiencies may be largely unlinked to the structural determinants like wealth and education in contrary to what the existing frameworks suggest, because the deficiency status may be attributable to inadequate uptake and absorption from Indian diets and therefore is substantially noticeable in every socio-economic stratum of India. Repeated infections may also contribute to this deficiency status through increased loss and inadequate utilization. It is worth mentioning in this context that genetic haemoglobinopathies are also posited as immediate determinants of anaemia, but one out of two Indian women experience anaemia whereas genetic disorders are much rarer [25]. Therefore, these haemoglobinopathies are likely to have limited contribution to this large anaemia burden in the Indian society.

The next part of our analysis showed that the most socially disadvantaged sub-population that is the tribal NPWRA experienced a 7.1-point anaemia “decline” between 2006 and 2016, when their general caste counterparts, the most socially advantaged group, did not experience any such decline during the same period. During other periods, that is between 1998 and 2006 and between 2016 and 2021, the tribal anaemia moved parallelly with that of the general, both showing modest increase in anaemia levels in those two periods. However, the changes in the socio-economic conditions of the tribals between 2006 and 2016 could only explain 23% of the 7.1-point decline in their anaemia. The majority of the unexplained portion of this decline may be attributed to the unprecedented reduction in malaria in India during that decade [43–45], because malaria affects mainly the tribal-dominated regions of India. But notably, even after the 7.1-point decline, anaemia rates persisted at > 50% among tribal NPWRA. It implies that perhaps initiatives like malaria control have limited impact on anaemia reduction in India, and drastic reduction in anaemia as per SDG goals can only be achieved when the main driver that is deficiency of micronutrients due to inadequate uptake and malabsorption are holistically addressed.

We re-examined the phenomenon of “decline” from a different angle, this time examining the “narrowing” of tribal/general anaemia gap during the same period. The decadal narrowing in the disparity of structural determinants could explain only 7% of the narrowing of anaemia – again exposing the limitation of the structural determinants in explaining anaemia trends in India. Therefore, our analysis goes well beyond the “beaten path” of highlighting the role of structural determinants in causing anaemia [46–49]. Unlike those cross-sectional studies that just highlighted the socioeconomic pattern of anaemia, we examined longitudinal trends of anaemia and its determinants, perhaps for the first time among tribals, and therefore could find that improvements in structural determinants did not explain the mostly stagnant anaemia trends. Therefore, we suggest that future research should focus on the tenacious persistence of absolute and functional iron and micronutrient deficiencies in all strata of Indians despite their burgeoning affluence and other social developments [49, 50] and this may require India-centric reframing of the well-known existing causal frameworks of anaemia. The history of public health programmes to combat anaemia in India is half a century old [29]. Almost all these interventions comprise iron-folate supplementation of pregnant and lactating women and children and adolescents, and few also focus on deworming children. A large proportion of the women population that is NPWRA, were not covered by these programmes [29]. But recently, anaemia programmes have been revised and expanded to target them with supplementation also [51, 52]. However, the roles of these programmes in reducing anaemia need critical review because unless the core issue of micronutrient uptake and absorption is not addressed, mere supplementation may not work as is the case currently. If India and the world along with it has to achieve 50% reduction in the current anaemia burden by 2030 as per the targets included in the SDGs; increased investment in novel aetiological research and new interventions in this field are imperative.

Most likely there is a need to bring about substantial changes in the quantity and quality of the Indian diet, in every socioeconomic stratum, so that intake and absorption of the micronutrients including iron and their utilization in manufacturing of adequate haemoglobin containing red blood cells in sufficient numbers can be ensured across the board. Different region-specific dietary interventions perhaps need to be designed keeping in mind the tremendous socio-cultural and dietary diversity of India. Fortification of cereals, staples and other food may be another critical channel of addressing the dietary micronutrient deficiency issue. However, good quality food fortification at scale for a country of 1.4 billion people may be a real challenge. Rapid reduction of diseases like Malaria and Tuberculosis through their existing control programmes can also contribute to the anaemia reduction initiatives in the country. Additionally, novel aetiologies of anaemia such as air pollution as posited by some Indian researchers should also be further explored [53].

Our study has a few limitations. First, some of the determinants explaining anaemia burden could not be included in the study such as self-reported data of iron folic acid tablets or syrups distribution and its compliance, as the data was available only for a small subset of women currently pregnant or had given birth in the last three years (NFHS-2, 3) or five years (NFHS-4, 5) preceding the survey. Similar was the data availability situation for access to maternal health care and information related to food consumption of women. These variables could not be included for the risk of underpowering our analysis by truncating the sample size. But this does not undermine our study as our main aim was to explore the role (or its absence) of structural determinants (*underlying* and *intermediate*) in explaining the trends of anaemia in India. Second, 5.1% of tribal/general NPWRA (*n* = 21,287) had to be excluded from our analysis as their haemoglobin data were missing. The missing group was richer, more privileged and urban-dwelling, hence less likely to be anaemic. However, their exclusion is unlikely to bias our results as we mainly examined within-group trends and between-group differences and not overall pooled estimates (if our analysis would have involved pooled estimates, then the exclusion of a privileged ‘missing’ group would have introduced some bias towards overestimation of anaemia). Lastly, the projected estimates of NFHS-5 may be affected by the interruption of the health services due to the current COVID-19 pandemic. However, the significant strength of our study lies in the use of four nationally representative datasets of NPWRA, spanning over 23 years, with objectively measured outcomes and determinants.

## Conclusion

To conclude, our study has shown decline in anaemia in the most disadvantaged sub-population of India, tribal NPWRA, between 2006 and 2016 (however this sub-population did not display any decline of anaemia at any other period between 1998 and 2021), in the backdrop of constant high-level or even increasing anaemia burden in the most advantaged group that is general caste NPWRA. However, the tribal/general disparity in anaemia was present throughout, but with discernible narrowing between 2006 and 2016, attributable to the decline in tribal anaemia during this period. This scenario was despite remarkable improvement in socio-economic situation of the both the groups during the 23-year study period. Meanwhile, only a small portion of this “decline” or “narrowing” could be explained by temporal changes in the structural determinants, signifying the drivers of anaemia among Indian women may be well beyond these socio-economic factors and their inequalities. Therefore, our study adds to the body of evidence that the main drivers of anaemia in India may be absolute and/or functional deficiency of micronutrients including iron, emanating from inadequate uptake and absorption of these elements from Indian diets, and these are largely unlinked to the structural determinants. Therefore, future aetiological and implementation research for anaemia reduction in India should focus on these drivers.

## Data Availability

The datasets generated and/or analysed during this work are accessible in the International Institute for Population Sciences’ repository in Mumbai, India, and can be viewed freely available in public domain through https://www.iipsindia.ac.in/

## Abbreviations

NPWRA: Non-Pregnant Women of Reproductive Age Group
NFHS: National Family Health Survey
WHO: World Health Organization
Hb: Haemoglobin
g/dL: Grams (g) per deciliter (dL)
WRA: Women of Reproductive Age
WHA: World Health Assembly
GNT: Global Nutrition Target
UNICEF: United Nations Children's Fund
UN: United Nations
SDG: Sustainable Development Goal
ST: Scheduled Tribes
BMI: Body Mass Index
USD: United States dollar
